# Histopathological Features of Gerhardt Syndrome in a Patient With Multiple System Atrophy: An Autopsy Case Report

**DOI:** 10.7759/cureus.30415

**Published:** 2022-10-18

**Authors:** Keiichi Nakahara, Koutaro Takamatsu, Noritaka Kudo, Takaaki Ito, Mitsuharu Ueda

**Affiliations:** 1 Neurology, Kumamoto University, Kumamoto, JPN; 2 Pathology, Kumamoto University, Kumamoto, JPN

**Keywords:** group atrophy, posterior cricoarytenoid muscle, pathological autopsy, gerhardt syndrome, multiple system atrophy

## Abstract

Multiple system atrophy (MSA) is a progressive neurodegenerative disease characterized by autonomic failure, parkinsonism, and cerebellar ataxia. Gerhardt syndrome, which is inspiratory dyspnea with laryngeal stridor associated with dysfunction of the vocal folds, is a frequent and fatal complication of MSA. A 59-year-old man with a six-year history of MSA presented with ataxia and dysarthria. He also had dyspnea and stridor, which had worsened in the last three months, and died from respiratory distress. Autopsy revealed neurogenic group atrophy of the posterior cricoarytenoid (PCA) muscle, which suggested that laryngeal nerve damage caused abductor vocal fold paralysis in addition to cerebellar and brainstem atrophy with glial cytoplasmic inclusions. Our histopathological findings suggest that Gerhardt syndrome may be associated with neurogenic atrophy of the laryngeal abductor muscle (PCA muscle) of the vocal folds.

## Introduction

Multiple system atrophy (MSA) is a progressive neurodegenerative disease characterized by a variable combination of autonomic failure, parkinsonism, and cerebellar ataxia, which are pathologically associated with oligodendroglial aggregation of α-synuclein and neuronal loss, predominantly in the striatonigral and olivopontocerebellar systems [1]. MSA rapidly progresses with a mean survival from onset of eight to ten years. Gerhardt syndrome, which is inspiratory dyspnea with laryngeal stridor associated with dysfunction of the vocal folds, is a frequent and fatal complication of MSA [2-4]. Although several causal hypotheses such as laryngeal abductor muscle weakness and dystonia in laryngeal adductor muscles have been proposed [4], the pathological bases of Gerhardt syndrome in MSA remain controversial. Herein, we report histopathological findings of an autopsied MSA case with Gerhardt syndrome.

## Case presentation

**Figure 1:**
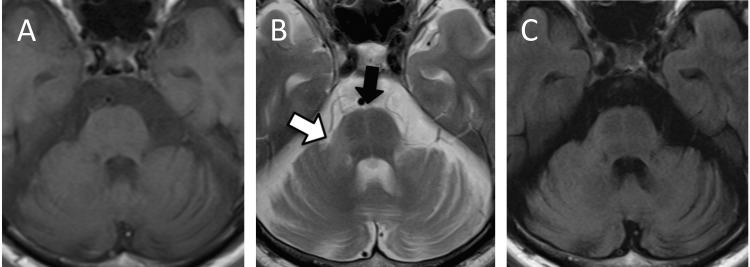
Brain MRI findings Brain MRI shows atrophy of cerebellar and middle cerebellar peduncles (MCP), enlargement of the fourth ventricle, hyperintensity in the MCP (MCP-sign) (white arrow), and hot cross bun sign (black arrow). A: Axial T1-weighted MRI, B: Axial T2-weighted MRI, C: Fluid attenuated inversion recovery (FLAIR)

A 59-year-old man was aware of his dysarthria at 52 years of age. Gradually his dysarthria progressed and he became prone to falls, so he presented at 53 years of age. He also had progressive cerebellar ataxia, urinary frequency, erectile dysfunction, and stridor at night. He had no medical history and no medications. The family medical history was not relevant. Neurological examination revealed scanning speech, left upper limb rigidity, limb ataxia, and orthostatic hypotension. All other clinical findings associated with the cranial, motor, and sensory nerves, as well as reflexes, were normal. Brain magnetic resonance imaging (MRI) revealed atrophy of the cerebellar and middle cerebellar peduncles (MCP), enlargement of the fourth ventricle, hyperintensity in the MCP (MCP sign), and hot cross bun sign (Figure 1). 

**Figure 2:**
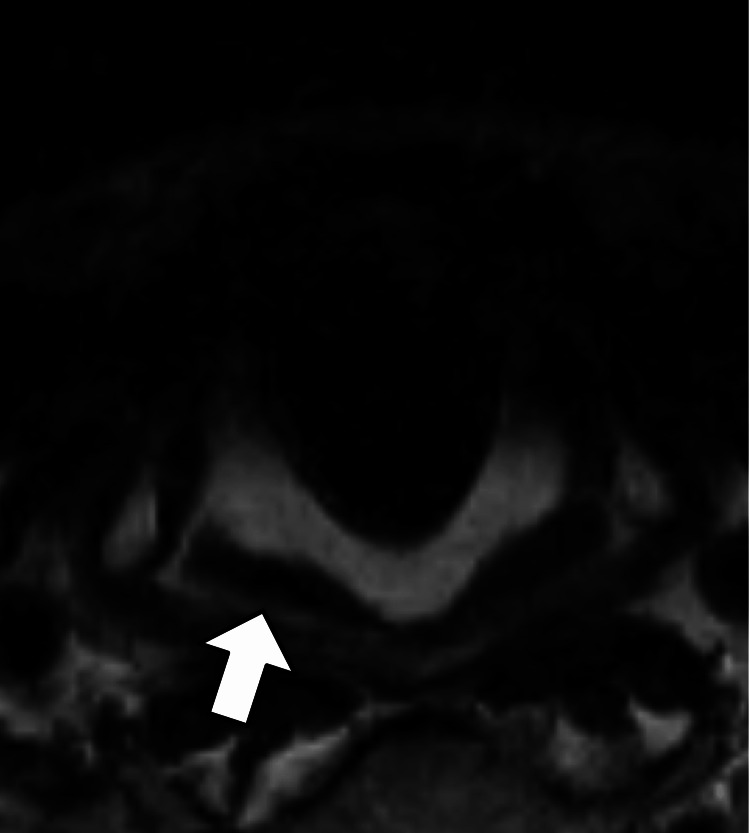
Laryngeal MRI finding Axial T2-weighted MRI on initial admission shows no atrophy of the posterior cricoarytenoid (PCA) muscle (arrow).

N-isopropyl-p-[123I] iodoamphetamine (IMP) single-photon emission computed tomography (SPECT) revealed decreased perfusion on the cerebellar hemispheres. Dopamine transporter (DAT) SPECT revealed decreased accumulation of bilateral putamens. Based on these clinical findings, he was diagnosed with MSA. No obvious atrophy of the posterior cricoarytenoid (PCA) muscle was found on MRI (Figure 2).

He was prescribed taltirelin hydrate, which was ineffective and was discontinued. He needed a walker to get around at 54 years of age. His dysarthria worsened, he became unable to communicate, and he began choking while eating at 55 years of age. The symptoms continued to progress.

**Figure 3:**
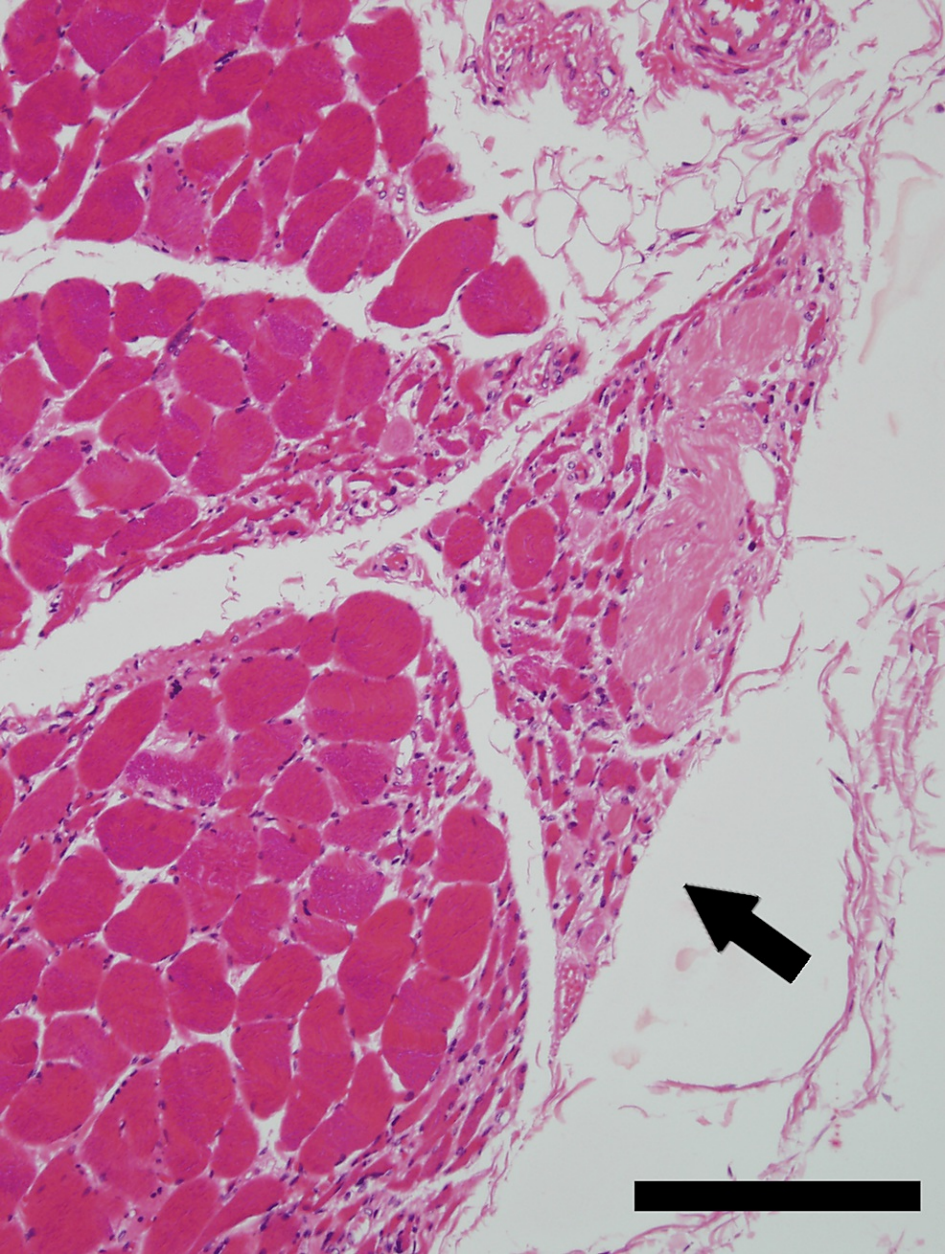
Neurogenic group atrophy of the posterior cricoarytenoid muscle in an autopsy case with multiple system atrophy (MSA) Neurogenic groups atrophy of the posterior cricoarytenoid muscle (arrow). Hematoxylin and eosin, scale bar 200 µm.

His dyspnea and stridor worsened over the last three months. He was admitted to our hospital with respiratory distress and altered consciousness. Neither he nor his family wanted ventilator or other life-support treatments and he died. An autopsy study revealed phosphorylated alpha synuclein-positive glial cytoplasmic inclusion in the cerebellum, pons, and inferior olivary nucleus. Furthermore, it revealed neurogenic group atrophy of the PCA muscle, which suggested that his inspiratory dyspnea with laryngeal stridor was caused by abductor paralysis of the vocal folds associated with laryngeal nerve dysfunction (Figure 3).

## Discussion

Laryngopharyngeal dysfunction is strongly associated with decreased survival of patients with MSA [5]. In laryngeal endoscopic studies, laryngeal movement disorders, such as irregular arytenoid cartilages movements, paradoxical vocal fold motion, vocal fold motion impairment, and vocal fold fixation were reportedly found in patients with MSA in association with inspiratory stridor [2-4]. Several causal hypotheses of Gerhardt syndrome in MSA have been proposed [4]. One of them is dystonia of the laryngeal adductor muscle and another is the weakness of the laryngeal abductor muscle. Electromyography using a needle electrode showed abnormal persistent tonic activity in the adductor muscles during sleep in patients with MSA with laryngeal stridor [6]. Botulinum toxin injection into the adductor muscle determined subjective improvement and reduced tonic EMG activity [7]. These results support the dystonia of the laryngeal adductor muscle.

In our autopsy case, MRI did not show any evidence of atrophy of the laryngeal abductor muscle of the vocal folds (the PCA muscle), but laryngeal histopathological findings suggested that his Gerhardt syndrome was associated with neurogenic atrophy of the PCA muscle. Our findings were consistent with several other autopsy studies with a limited number of cases (total of 15 cases) [8-10]. Pathophysiologically, serotonergic neurons in the nucleus raphe pallidus are thought to provide tonic excitatory drive to the PCA motor neurons via 5-HT2 receptors [11]. Depletion of medullary serotonergic neurons has been observed in the brains of patients with MSA who succumbed to sudden death [12]. Thus, depletion of medullary serotonergic neurons may lead to impairment of nerve fibers innervating the PCA muscle in patients with MSA. 

On the other hand, another EMG study showed contraction of the adductor muscle and gradual relaxation of the abductor muscle during inspiration, especially during sleep [13]. From these findings, it was inferred that dystonia of the adductor muscle and weakness of the abductor muscle coexist in Gerhardt’s syndrome, rather than being in opposition to each other. Future studies combing laryngeal EMG and histopathological evaluation are desirable.

## Conclusions

Our patient with MSA died from inspiratory dyspnea with laryngeal stridor associated with dysfunction of the vocal folds at six years after onset of the disease. Gerhardt syndrome, a frequent and fatal complication of MSA, may be associated with neurogenic atrophy of the laryngeal PCA muscle.
